# Recurrent Spiking Neural Network Learning Based on a Competitive Maximization of Neuronal Activity

**DOI:** 10.3389/fninf.2018.00079

**Published:** 2018-11-15

**Authors:** Vyacheslav Demin, Dmitry Nekhaev

**Affiliations:** ^1^National Research Center “Kurchatov Institute”, Moscow, Russia; ^2^Moscow Institute of Phycics and Technology, Dolgoprudny, Russia

**Keywords:** spiking neural networks, unsupervised learning, supervised learning, digits recognition, classification, neuron clustering

## Abstract

Spiking neural networks (SNNs) are believed to be highly computationally and energy efficient for specific neurochip hardware real-time solutions. However, there is a lack of learning algorithms for complex SNNs with recurrent connections, comparable in efficiency with back-propagation techniques and capable of unsupervised training. Here we suppose that each neuron in a biological neural network tends to maximize its activity in competition with other neurons, and put this principle at the basis of a new SNN learning algorithm. In such a way, a spiking network with the learned feed-forward, reciprocal and intralayer inhibitory connections, is introduced to the MNIST database digit recognition. It has been demonstrated that this SNN can be trained without a teacher, after a short supervised initialization of weights by the same algorithm. Also, it has been shown that neurons are grouped into families of hierarchical structures, corresponding to different digit classes and their associations. This property is expected to be useful to reduce the number of layers in deep neural networks and modeling the formation of various functional structures in a biological nervous system. Comparison of the learning properties of the suggested algorithm, with those of the Sparse Distributed Representation approach shows similarity in coding but also some advantages of the former. The basic principle of the proposed algorithm is believed to be practically applicable to the construction of much more complicated and diverse task solving SNNs. We refer to this new approach as “Family-Engaged Execution and Learning of Induced Neuron Groups”, or FEELING.

## 1. Introduction

Compared to formal neural networks, spiking neural networks (SNNs) have some remarkable advantages, such as the ability to model dynamical modes of network operations and computing in continuous real time (which is the realm of the biological prototype), the ability to test and use different bio-inspired local training rules (Hebb's, Spike-Timing Dependent Plasticity (STDP), metabolic, homeostatic, etc.), significantly reduced energy consumption of SNNs realized in specific multi-core hardware (neurochips) (Merolla et al., 2014), and others. Despite the great work done so far in the field of SNN learning, there is still a lack of effective algorithms comparable in perfomance, to formal neural network training techniques. Moreover, there is still the big challenge “How do our brains learn without a teacher?,” the answer to which has been sought for decades.

The traditional ways of SNN parameters setting are (i) the transfer (adaptation) of parameter values of a formal neural network to an SNN with the same (or similar) architecture (Diehl et al., 2015), (ii) the adaptation of learning algorithms suitable for the formal neural networks, such as the back-propagation, to SNN (Lee et al., 2016), and (iii) the training based on the bio-inspired local inter-neuron rules, such as STDP, of SNN with a biologically plausible architecture, e.g., with competition between neurons in Winner-Takes-All (WTA) networks (Diehl and Cook, 2015).

The first approach is appealing due to a wealth of experience accumulated in the field of formal network training, with the use of back-propagation techniques, which minimize the value of some loss function with different weight update rules (SGD Bottou, 1998, Nesterov momentum Sutskever et al., 2013, Adagrad Duchi et al., 2011, Adadelta Zeiler, 2012, Adam Kingma and Ba, 2014). At the same time, the transfer of parameters determined by elaborate training methods from a formal to spiking network, is not a trivial task. The accuracy of problem solution can be reduced, and a special technique, usually specific to the task and/or architecture used, should be applied (Diehl et al., 2015).

The second approach tries to adopt the most developed back-propagation learning algorithms for direct use in networks with spiking neurons. Besides a specificity with respect to a spiking neuron model used, this method can be (limitedly) applied only to feed-forward SNN. Nevertheless, gradient-based methods still remain the state-of-the-art approach for training SNN (Lee et al., 2016).

The third way is still under development and therefore not perfect. At present, the use of local training rules cannot compete with the well-established industrial algorithms of learning formal neural networks to solve different practical tasks. Nevertheless, this approach has a great potential for the coming generation of SNN algorithms for intelligent information processing. This is due to (i) the ability to develop new bio-inspired self-learning methods (not requiring the huge amount of labeled data), (ii) the ability to build up and train complex SNN architectures with recurrent connections, cross-linked association, reinforcement, attention, and other types of neuronal layers, and (iii) the capability to realize the real-time, energy-efficient information processing systems with non-linear dynamics corresponding to different applications (communications, household appliances, industrial production, robotics, etc.) on the base of special neuromorphic hardware. There is of great interest and many studies describing the first results of developing local training rules for the SNNs (Izhikevich, 2007; Legenstein et al., 2008; Lazar et al., 2009; Clopath et al., 2010; Querlioz et al., 2013; Diehl and Cook, 2015; Zhao et al., 2015; Kheradpisheh et al., 2017; Sanda et al, 2017; Sboev et al., 2017, 2018; Mozafari et al., 2018), on the one hand, as well as many studies concerning the first steps in a hardware realization of neuromorphic computing systems with analog weights and a spiking architecture (Demin et al., 2015; Prezioso et al, 2015; Covi et al., 2016; Emelyanov et al., 2016; Serb et al., 2016; Wang et al, 2018), on the other hand.

A Particular case of developing local training rules is the modification of STDP that converts a task in to reinforcement learning. The weight change is modulated by a global reward signal that often has the meaning of increasing the dopamine concentration. This model, proposed by Izhikevich (2007), has been researched analytically by Legenstein et al. (2008), and this work continues to date (Kappel et al., 2017). This approach showed an ability to memorize temporal spike patterns (Legenstein et al., 2008) and solved the problem of finding a forage by a robot (Sanda et al, 2017). Timothee Masquelier (Mozafari et al., 2018) created a model that solves the MNIST benchmark with a multilayer network that has dopamine-modulated STDP applied to the last layer.

Another direction of research is reservoir networks. Maass has introduced the Liquid State Machine (Maass et al., 2002), which is a large neural network with random connections, where hidden neurons apply a lot of nonlinear transformations to the input data. He showed analytically that, if the number of neurons are large enough, the classifying layer can learn to reproduce any desired output. The classifying layer can be trained with a genetic algorithm (Schliebs and Kasabov, 2013), for example. Liquid State Machines were also used with various kinds of plasticity, including STDP, so the model showed better performance in memorizing spatio-temporal patterns than the Liquid State Machine with static weights (Lazar et al., 2009).

Claudia Clopath has introduced a kind of synaptic plasticity based on mechanical principles and investigated its behavior in small networks in relation to the type of the input signal: temporal or rate-coded. It was shown that for temporal coding of signals (Clopath et al., 2010) the FORCE learning rule (Sussillo and Abbott, 2009) was useful to learn various oscillatory behaviors, to solve classification tasks and even to reproduce a bird song (Nicola and Clopath, 2017). The work has demonstrated the importance of high dimensionality of the input signal, which increases the effectiveness of the FORCE learning method.

All the results shown are only paving the way to the above-mentioned task of elaborating an effective SNN learning algorithm based on the local inter-neuron relations. So, in this work, we aim to make the next step, suggesting a simple universal principle for the development of local training rules of the BCM-like type (Bienenstock et al., 1982), responsible for a weight update between spiking neurons of different functionality (excitatory or inhibitory).

The neuron model, recurrent SNN architecture chosen and some other implementation details will be described in section 2.1. The bio-inspired premises, basic assumptions and suggested local training rules will be discussed in section 2.2 of the article. Section 3 demonstrates the main results of the application of our rules for training the special SNN architecture to solve the bench-marking task of MNIST database handwritten digit recognition, both supervised and partially unsupervised. The brief analysis based on the weights clustering and visualization shows that families (or groups) of neurons corresponding to different classes of digits, are formed in the processes of competition and cooperation between neurons during training. So, we refer to the new algorithm as ‘Family-Engaged Execution and Learning of Induced Neuron Groups', or FEELING. In section 4, we compare our algorithm with the Sparse Distributed Representation approach, discuss some perspectives of the FEELING algorithm, its further development and usage.

## 2. Materials and methods

### 2.1. SNN architecture and implementation details

#### 2.1.1. SNN architecture

Here we consider a simple SNN architecture with 2 layers of neurons, which is sufficient for the demonstration of the proposed FEELING algorithm with the MNIST benchmark. Considerations about using this algorithm for more complex network architectures will be outlined in section 2.3.

**Figure 1:**
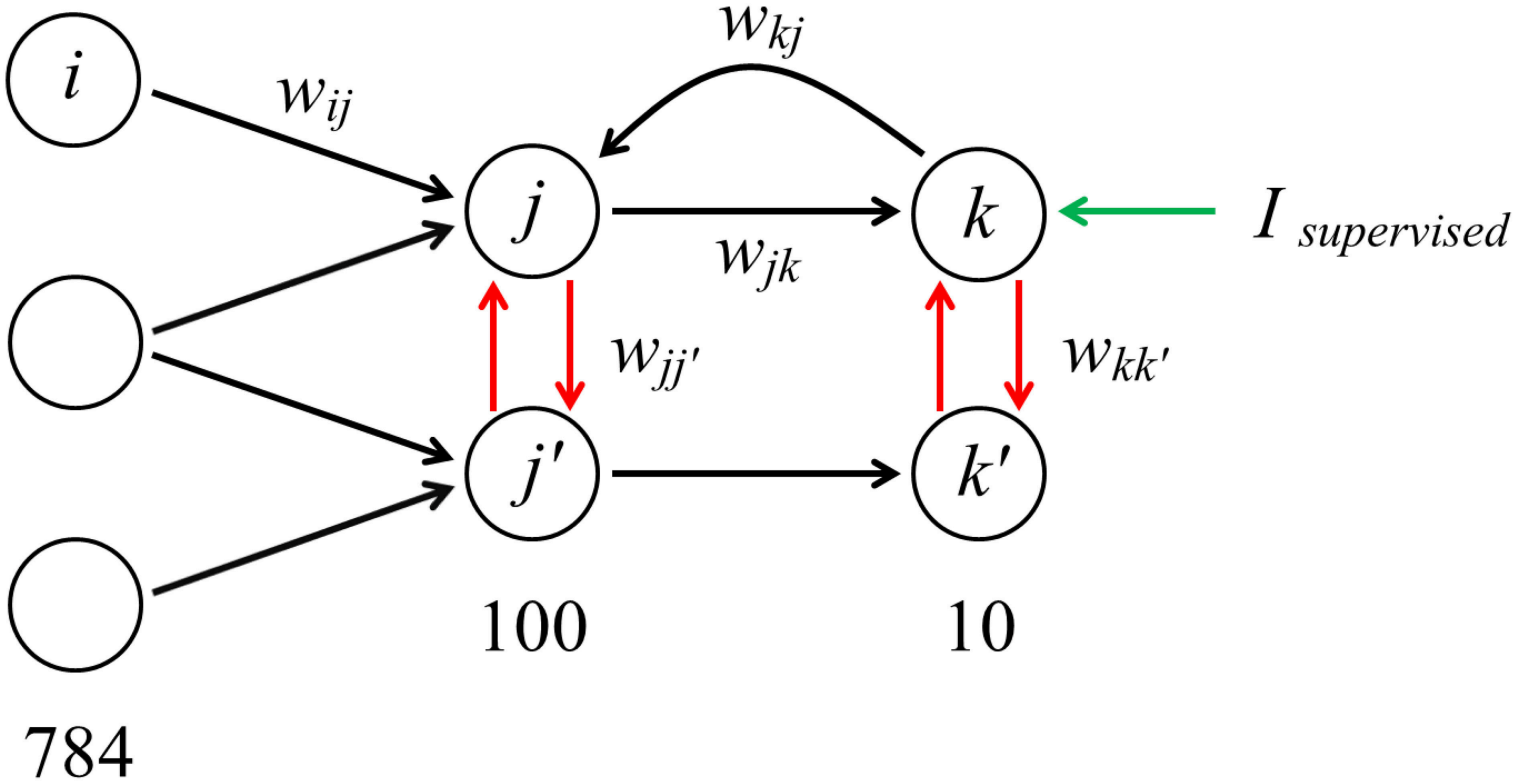
“784−100−10” architecture of the model with forward, lateral and reciprocal connections (the architecture is fully interconnected; only a few connections are shown). When training the network, an additional supervised current can be introduced into the neuron corresponding to image of a certain class presented at the input.

We constructed the SNN in the architecture “784−100−10” (784 neurons in the input layer, 100 in the hidden layer and 10 classifying neurons in the output layer). There are three types of connections between neurons. The first one is the standard feed-forward connections (from input to hidden, and from hidden to output layer), whose weights are in the range of 0 to 1. The second is the negative lateral connections (from –1 to 0) that model inhibitory connections between neurons inside the layer and helps to learn faster. The third one is reciprocal connections from the output layer directly to the neurons of the hidden layer. This architecture is presented in Figure 1.

#### 2.1.2. Activity calculation

**Figure 2:**
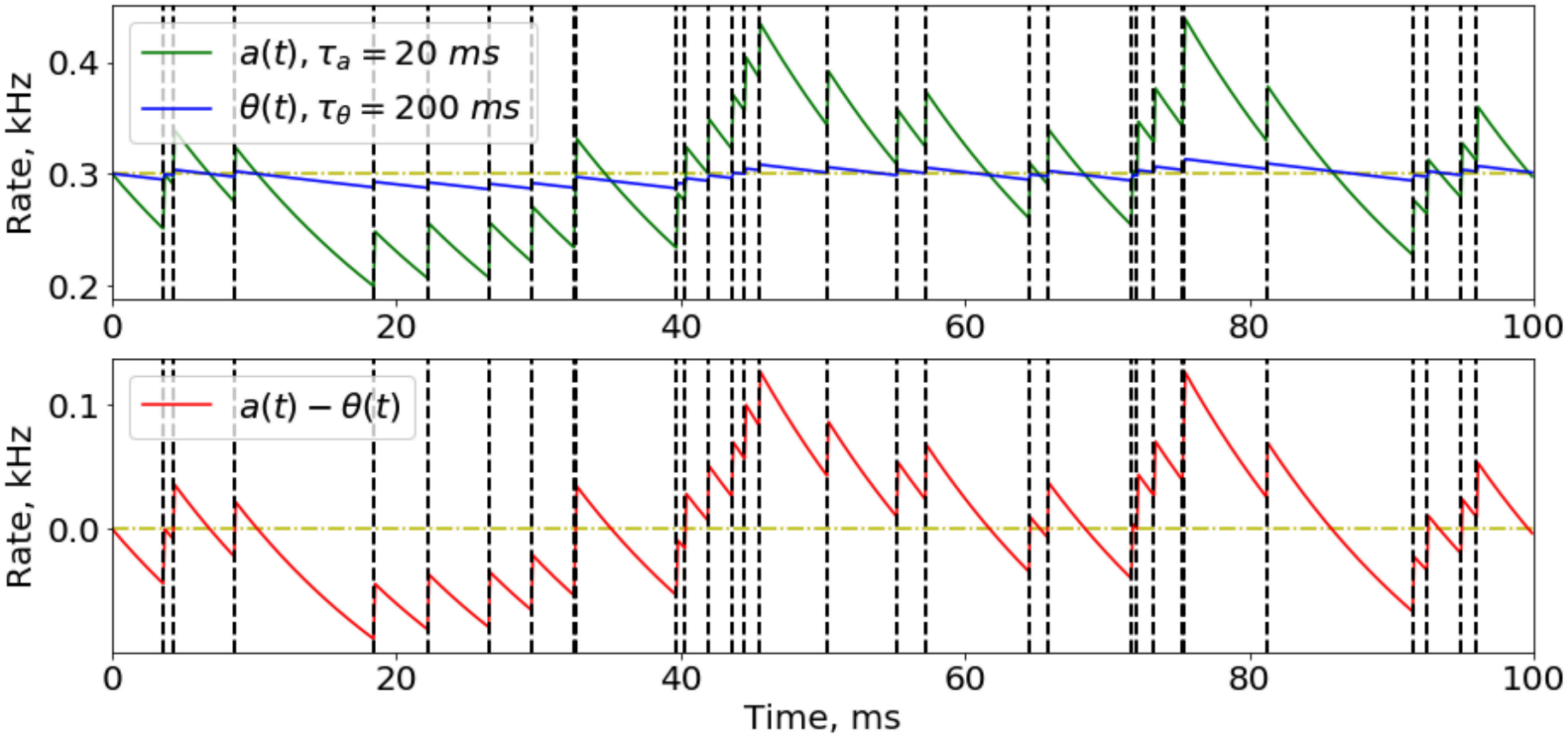
Approximation of instant and average neuron activity. Instant (green line) and average (blue line) firing activities are calculated as the Exponential Mean Average of the spike time-series. Spike train here was obtained from a Poisson distribution with firing probability of 0.3 at every time step (300 Hz rate). The difference between instant and average firing rates while presenting a Poisson-distributed input signal with constant probability has a noisy effect on the network training, because most of update rules depend on this difference. Moments of spikes are highlighted by vertical dashed lines.

An important part of implementing our training rules in the SNN, is to efficiently compute the instant and average firing activity for all the neurons at every time step. The solution is that we do not really need an accurate value of the activity (which is a sum of all action potential amplitudes multiplied by their relaxation kinetics), so we can just find a good approximation that is easy to calculate. If we consider a spike train as a time-series of 0's and 1's, where 1 corresponds to the moment of spike, its Exponential Mean Average (EMA) could be a good solution for approximating the firing activity (Figure 2) for time intervals of different lengths, for *a* and θ, which we will call “instant” and “average” activity, respectively:

(1) $$\begin{array}{l} a\left(t\right)=\left(1-\frac{\Delta t}{\tau_{a}}\right)a\left(t-\Delta t\right)+\frac{s\left(t\right)}{\tau_{a}}, \end{array}$$

(2) $$\begin{array}{l} \theta \left(t\right)=\left(1-\frac{\Delta t}{\tau_{\theta }}\right)\theta \left(t-\Delta t\right)+\frac{s\left(t\right)}{\tau_{\theta }}. \end{array}$$

Here, τ_*a*_ and τ_θ_ are the hyper-parameters to be chosen (τ_*a*_≪τ_θ_), Δ*t* = 1 ms is the simulation time step, and *s*(*t*) is a binary spike variable equaling 1 if spike occurs at the moment *t*, and 0 otherwise. As a first iteration for adjusting these time constants it is suitable to set $\tau_{\theta }=\frac{T_{input}*n}{5}$, where *T*_*input*_ is a time necessary for presenting one image (see also sections 2.4, 2.5) and *n* is the number of image classes in a classification task (for the MNIST database this is 10); τ_*a*_ should be reasonably set to the value $\frac{T_{input}}{5}$ or less. It is worth noting that once the parameters τ_*a*_ and τ_θ_ are chosen, an interesting feature arises. It concerns the non-zero firing rate fluctuations around the average value of (*a*−θ) difference, even after a long period of time, enough for the full relaxation of the neuron to its stationary state. These fluctuations can be considered as noise added to the input signal of the neuron. Some considerations on the role of fluctuations in the learning are presented in Supplementary Material, section 1.

It is important to note that the addition of instant and average firing rates to the model of the neuron, does not make the implementation of the neuron in a neurochip too complicated, because the activity is calculated similarly to the leaky threshold potential with a different time constant. The implementation of the LIF neuron model with the leaky threshold model has been shown in Indiveri et al. (2011).

#### 2.1.3. Neuron model

During the simulation we used a leakage-integrate-and-fire (LIF) neuron model (Burkitt, 2006) to define the dynamics of the membrane potential of the neuron. When the potential *V*_*j*_ rose to the threshold value with a characteristic membrane time τ_*m*_ = 20 ms, the neuron generated a spike and entered the period of refractoriness with τ_*ref*_ = 2 ms:

(3) $$\begin{array}{l} \tau_{m}\frac{dV_{j}}{dt}=-V_{j}\left(t\right)+I_{j}\left(t\right). \end{array}$$

The input current of a post-synaptic neuron *j* was calculated as a weighted sum of instant activities of all pre-synaptic neurons:

(4) $$\begin{array}{l} I_{j}\left(t\right)=\underset{i}{\sum }w_{ij}a_{i}\left(t\right). \end{array}$$

This neuron model was used in all the layers except the input layer. The inputs at the first layer were simulated by the Poisson-distributed spiking trains with the average rates proportional to the input signal level (the pixel intensities). This allowed saving computation time to process every neuron in the input layer.

For the sake of equitable competition and correct training of spiking neurons, an adaptive dynamic threshold was used that depends on the neuron firing rate (this property is bio-inspired as it was observed for the real neurons in the brain Zhang and Linden, 2003). The easiest way to implement this was to set the threshold as an exponentially decaying value with the time constant τ_*th*_ (50 ms in our case) and increasing it abruptly at the moment of a spike by a constant that depends on the number of neurons *N* in the corresponding layer:

(5) $$\begin{array}{l} \frac{dV_{th,j}}{dt}=\Delta V_{th}N\delta \left(t-t_{j}\right)-\frac{V_{th,j}}{\tau_{th}}, \end{array}$$

where Δ*V*_*th*_ = 0.1 is the increment for the threshold.

For all the simulations of the SNN training and functioning the freely distributed software BRIAN was used (Goodman and Brette, 2008).

### 2.2. Bioinspired training rules

To propose a universal principle for the interneuron synaptic plasticity which determines the local training rules for artificial SNN we have to ponder the question: “What could be the purpose, or the evolution–designed mission, of each individual neuron's life in a biological neural network?” It is well known that adult neurons do not have the capability of division, so reproduction cannot be the purpose of a neuron. On the other hand, it is involved mostly in information processing which, from the biological point of view, means the generation of action potentials by the neural cell. So it is reasonable to suppose that every neuron tends to support its activity at a high level to justify its biological role in the whole network. We propose to go further and assume that a neuron striving to maximize its activity in competition with other neurons, is a good premise for the SNN learning algorithm.

This hypothesis is inspired by the following biological observations. First of all, there are many studies about the so-called synaptic pruning, which means elimination of the neuron's synaptic connections, mostly during the developmental stage of an organism (Chechik et al., 1998, 1999; Iglesias et al., 2005; Vanderhaeghen and Cheng, 2010; Petanjek et al., 2011). Generally, it is associated with the competition of synapses for the neurotrophic factors. Only those get the sufficient survival resources that justify their biological role of transmitting a strong signal to a cell, likely consistently (in conjunction) with input signals through the other synapses of this neuron.

Second, there is a lot of evidence for the neuronal programmed cell death (mainly, apoptosis) during the experience-free pre-natal and the experience-dependent early development of organisms, from insects to vertebrates and humans (Mazarakis et al., 1997; Abitz et al., 2007; Fuchs and Steller, 2011; Kristiansen and Ham, 2014; Yamaguchi and Miura, 2015). It has been assumed that this mechanism is not only due to the necessity of the elimination of damaged or stochastically degraded cells, but is also very important for learning, i.e., continually maintaining more efficient brain functions by removing neurons because of their information processing inefficiency (Chechik et al., 1999). It is well known that adults have significantly fewer neuron populations in some regions of the brain, e.g., in the mediodorsal thalamic nucleus (Abitz et al., 2007), than newborns. It is believed that this is due to an initial surplus of neurons which is competitively removed from the brain during experience-based development, so only cells that correspond to important behavioral and functional properties remain.

Third, there is some circumstantial support from the experiments *in vitro* on learning in a culture of real cortical neurons (Shahaf and Marom, 2001; Bakkum et al., 2008), when repetitive electric stimulation of a network drives a change of its synaptic connectivity pattern until stimulus is removed or modified. It may be a sign of the network reconstructing its connections due to the appearance of a group of highly active neurons under repetitive input. Other cells try to make or strengthen the connections to those leading neurons and become highly active too (secondary activity). Then the remaining neurons establish synapses with the secondary activity cells, and so on along the chain. This may be the mechanism of continuous reconstruction of the network topology and obtaining almost any desired output pattern, as mentioned in the works on learning neuronal cultures (Shahaf and Marom, 2001; Bakkum et al., 2008). Of course, it is speculative until it is shown directly, but this is one of the simplest interpretations of how it works. It is indirectly confirmed by the absence of target learning under the application of stochastic spatio-temporal stimuli to a network, when neurons change their activity in a random way (Bakkum et al., 2008). It should also be specified that we talk about the signals and stimuli significantly below the physiological limit of neuron damage.

It is worth noting that additional, more specific studies are needed to firmly establish or correct the principle of competitive maximization of the activity of neurons, but we take it as a good bio-plausible working hypothesis to derive the new learning algorithm for SNNs.

Maximizing its activity, each neuron also reaches other related aims such as (i) maximizing its lifetime, (ii) increasing the level of trophic and biochemical energy resources, and (iii) an enhancement of representation of this neuron at the other neural layers. The second statement is due to the increased cell metabolism, and the third one is explained as follows. According to the described strategy, a particular neuron should increase weights with those neurons which have a relatively high activity at a given moment. So, keeping its activity high, the neuron guarantees that neural cells from the other layers seek to strengthen connections with it, thus enhancing the representativeness of that neuron at the other layers.

For our purposes, it is not important which specific aim, from those stated above, is the main one and which is secondary. From the practical point of view of SNN learning algorithm development, it is convenient to use the activity maximization principle as the basis. However, it is important to note that the maximization of neuronal activity needs the biochemical resources, which are actually limited at every moment. The neurons therefore compete with each other and re-distribute their current resources in such a way, as to obtain maximum input currents from other neurons. For example, a post-synaptic neuron should weaken the connection weights with low activity pre-synaptic neurons, in order to get an opportunity to increase other weights with more active pre-synaptic cells. This can be written as follows:

(6) $$\begin{array}{l} \frac{dw_{ij}}{dt}=\alpha \left(a_{i}-\theta_{i}\right)\delta \left(t-t_{j}\right)-\frac{w_{ij}}{\tau }. \end{array}$$

Here *a*_*i*_ determines the instant activity of a pre-synaptic neuron *i*, θ_*i*_ is the time-averaged activity of neuron *i* (see also section 2.1.2 for *a* and θ definition), α is a positive learning rate, δ stands for the Dirac delta-function corresponding to the moments of spikes of post-synaptic neuron *j*, and the $\frac{w_{ij}}{\tau }$ term means an exponential decay of the weight.

The weight update rule (6) can be motivated as follows. Post-synaptic neuron *j* strives to strengthen its connections with those pre-synaptic neurons with a higher than average activity, over some previous period of time and to weaken them otherwise. Of course, it is only one of the ways to express the idea of neuron activity maximization (due to increased connection weights with cells for which *a*_*i*_>θ_*i*_) under conditions of limited resources and competition between presynaptic neurons (due to decreased weights with cells for which *a*_*i*_ < θ_*i*_). A specific type of update rule could be quite different from that of (6), but it still has to reflect the activity maximization principle and to describe the economy and “forced uptake” of resources, including the competition between neurons.

Simple rules (6) can be implemented to train forward connections of an SNN, but not for reciprocal (from a logically deeper layer to those closer to the input) or lateral (intralayer, competitive) ones (see section 2.1.1 for the SNN architecture description). Note that the update rule (6) is event-driven as it is applied only at the moment of post-synaptic neuron spikes [the exponential decay of the weight in (6) is calculated according to an exponential factor dependent on the time difference between consecutive spikes]. Thus, this scheme is computationally economic.

To derive a rule for the backward connections *w*_*kj*_ update we can follow the same principle: a particular neuron of the most distant layer tries to maximize its activity and, in order to do this, provides an extra current to those neurons of the previous layer that are the most active at the moment and have strong positive forward connections *w*_*jk*_ to this neuron. This neuron therefore tries to increase the weights of its reciprocal connections to neurons with high activity in the previous layer:

(7) $$\begin{array}{l} \frac{dw_{kj}}{dt}=\beta \left(a_{j}-\theta_{j}\right)w_{jk}\delta \left(t-t_{k}\right)-\frac{w_{kj}}{\tau }. \end{array}$$

Here β is a positive learning rate for backward-type connections.

In both synaptic weight update rules described above it is assumed that the value of a weight is clamped between 0 and 1, so they are excitatory connections. In our model we also took into account the inhibitory connections inside a layer of the network, that provide the competitive interaction of neurons. It is a widely-used technique, but it is often implemented with the non-learnable WTA rule (Diehl and Cook, 2015). Here we describe the same idea, with the weight change based on the local competition of a pair of neurons in the last (classifying) layer of the SNN:

(8) $$\begin{array}{l} \frac{dw_{kk^{\prime }}}{dt}=-\gamma \left(a_{k}-\theta_{k}\right)\delta \left(t-t_{k^{\prime }}\right)-\frac{w_{kk^{\prime }}}{\tau }. \end{array}$$

Here γ is a positive learning rate for intralayer negative connections, and the weight values are clamped between –1 and 0. This update rule provides, obviously, a learnable competition. It should be noted that in this case the meaning of the term (*a*_*k*_ − θ_*k*_) is different from that of the rule (6) and (7). It roughly means that only a highly active neuron (i.e., more than on average and, consequently, has a high level of resources) can strengthen the competition with other neurons in this layer, and vice versa.

Finally, we would like to introduce not only competition, but also cooperation, inside the hidden layer. Some neurons that form a subgroup inside the hidden layer can simultaneously have high activities while processing the current input signal, so they start to cooperate to increase their lifetime and produce more powerful signals deeper into the network. Dynamics of this type of connection can be described as follows:

(9) $$\begin{array}{l} \frac{dw_{jj^{\prime }}}{dt}=\eta \left(a_{j}-\theta_{j}\right)\left(a_{j^{\prime }}-\theta_{j^{\prime }}\right)\delta \left(t-t_{j}\right)-\frac{w_{jj^{\prime }}}{\tau }. \end{array}$$

Here η is a positive learning rate for intralayer negative connections, and the weight values are also clamped between –1 and 0. This approach gives rise to subgroups, or families, of cooperating neurons inside the hidden layer, with small or zero negative weights between them (we consider cooperation as absence of competition). At the same time, these subgroups compete with each other, because neurons of different families tend to have strong negative connections with each other.

It is important to emphasize once again the update rules (6)–(9) are only one of the numerous ways to reflect the main biologically plausible principle of the spiking neuron: maximization of its activity in competition for resources. This, if correctly adjusted, guarantees the whole algorithm's convergence, in both supervised and unsupervised learning. The most complicated part of the rule design was the choice of specific formulae for the weight updates and hyper-parameter values such that an equitable competition between neurons was provided during the whole process of learning, especially in the initial phase. For the unsupervised training, a correct initialization of weights was also of crucial importance. If it is given by the uniform distribution, as in our case (see below), then the algorithm during the early training relies only on random similarities between the input vector and the weight vector of the neuron considered. In another case, if we train the neurons at the beginning of learning in the supervised manner, presenting to the SNN only a few labeled images, then the unsupervised training works much better in terms of algorithm's convergence, based on the same weight update rules. It will be demonstrated in section 3 of the article.

We do not claim that the particular kind of local training rules presented here is bio-inspired. Moreover, considering the formula (9) we postulated the existence of lateral inhibitory connections between excitatory neurons outside of the biological realm (in fact, the competition between excitatory neurons is mediated by inhibitory interneurons Faust et al., 2016). For us, compliance with the basic learning principle of the FEELING algorithm is the only relevant compliance. Nevertheless, we tried to select those rules in a simple form, similar to the plasticity of BCM type, which has some experimental evidence (Bienenstock et al., 1982) and is computationally admissible.

The similarity to the thoroughly explored BCM rule (Bienenstock et al., 1982) only refers to the use of the rate-coded instant and time-averaged variables *a* and θ and of their differences in type (*a* − θ). At the same time, the original BCM rule has a specific form for the weight modification in a feed-forward network between pre-synaptic neuron *i* and post-synaptic neuron *j*,

(10) $$\begin{array}{l} \frac{dw_{ij}}{dt}\propto a_{i}a_{j}\left(a_{j}-\theta_{j}\right)-\frac{w_{ij}}{\tau }. \end{array}$$

It differs from (6) by an additional factor of the pre-synaptic rate *a*_*i*_ (the multiplier *a*_*j*_ is approximately equivalent to the function δ(*t* − *t*_*j*_) accounting for the post-synaptic spikes) and by using the post-synaptic rate difference (*a*_*j*_ − θ_*j*_) instead of the pre-synaptic one (*a*_*i*_ − θ_*i*_) in (6). It is worth noting that the BCM rule (10) could also be used in the framework of the activity maximization principle, implying an increase in weights with active pre-synaptic elements if the post-synaptic neuron has enough resources, and vice versa. Regarding our experiments, the presence or absence of a factor of *a*_*i*_ was not critical for the training, but the use of the pre-synaptic difference, as opposed to (10), was important. The learning could not effectively converge by using the rule (10), because of the formation of a neuron group that captured almost all the image classes and suppressed other neurons in the layer. Presumably, this is due to a low image contrast training by the BCM rule compared to rule (6) (see Supplementary Material, section 2).

In addition to the remark in subsection 2.1.2 about *a* and θ emulation in hardware, it should be noted that the differences of the type (*a* − θ) can be realized either by specific differential amplifiers on chip and transforming it into a weight change control signal, or directly by a specific type of STDP. Indeed, Izhikevich has shown that the BCM rule is equivalent to the classical additive STDP taking place in a Poisson sequence of uncorrelated pre- and post-synaptic spikes (Izhikevich and Desai, 2003). Our rule (6), a non-essential factor of up to *a*_*i*_, may be derived in the same way as Izhikevich has done, but considering the post-synaptic instead of pre-synaptic centered spike pairing scheme. We also believe that other BCM-like weight modifications can be reduced to some special cases of STDP. In turn, different types of STDP were demonstrated in various memristive devices (Linares-Barranco and Serrano-Gotarredona, 2009; Prezioso et al., 2016; Pedretti et al., 2017; Lapkinab et al., 2018). It could therefore be the way to realize the proposed training scheme in a direct, on-line weight update manner. Further investigation however, is needed.

The values of hyperparameters used in the simulation are presented in Table 1. It should be noted that the model also uses additional hyperparameter coefficients for balancing the input currents of a neuron. The fact is that every neuron presents a non-linear transformation of the input signal. So, the signal is attenuated at the output of the neuron, and the feed-forward, inhibitory and reciprocal currents should be multiplied by some carefully chosen coefficients, to equalize their effects on the neuron. This task is considered in Supplementary Material, section 3.

**Table 1:** Values of hyperparameters used in the simulation.

**Hyperparameter**	**Value**
τ_*m*_	20 ms
τ_*a*_	15 ms
τ_θ_	150 ms
τ_*th*_	50 ms
τ_*ref*_	2 ms
τ	10 s
α	0.1
β	0.3
γ	0.1
η	0.15
Δ*V*_*th*_	0.1
*I*_*supervised*_	30

### 2.3. Scalability of the network's architecture

In general, the proposed principle and the corresponding rules based on that principle should work with any complex architecture of a recurrent SNN for any classification task, i.e., an SNN can be trained up to some appropriate recognition accuracy. The reason for this is that the neuron activity maximization principle relies on the competition between neurons responsible for different kinds or classes of input signal features, and works with any type of connections. However, there is a possibility that the convergence of learning is not achieved when some prevailing quantity of neurons forms a strong family, because of their cooperation, which suppresses the activity of all the other neurons. It depends mainly on the global and local neuron and the training rule parameters, which in current realization should be carefully adjusted for a chosen architecture. It might be promising to use some additional techniques for parameter space reduction and recognition accuracy improvement such as batch normalization or the metabolic rule defining a certain limit for the total excitatory weight sum of a neuron. The crucial necessity of cooperation and other methods to improve the whole algorithm will be discussed later in section 4.

There is also a question about the systematic choice of the weight update rules for more complicated SNN architectures. Despite the wide freedom of choice mentioned earlier, the local learning rules are constrained by the neuron activity maximization principle, on the one hand, and by the functionality of the neuron layers, on the other hand. If one would like to scale up a network, we should use the rules describing excitatory weight updates for the feed-forward and reciprocal connections of the types (6) and (7) for the repetitive hidden layers, respectively, and inhibitory weight change for the lateral competitive connections similar to the formula (9). The last, classifying layer is somewhat different from the hidden neuron layers and from any biological prototype. There is no layer in which each neuron is responsible for only one class of images. Therefore, the local training rules should be special for this artificial layer, with strong competition between neurons, similar to that defined in the formula (8).

Furthermore, for more complex SNN architectures, the spatial sparsity of neuron connections should most likely be ensured to detect low-level simple features at the shallow layers and provide the sparsity of the information representation. Some additional considerations about this are presented in section 4.

### 2.4. MNIST dataset encoding

The size of the SNN input layer was chosen according to the MNIST dataset which contains 60,000 training objects and 10,000 test objects that are images of 28 × 28 pixel images of handwritten digits with labels from 0 to 9 (LeCun et al., 1998). Three thousand training images were used as the validation set. Each digit was fed to the input of the network for *T*_*input*_ = 100 ms of simulation time in the form of Poisson-distributed spike trains with firing rates proportional to the intensity of the pixels of the input images, followed by 100 ms of the empty signal to allow the current and activity of neurons to decrease. The firing rates of the input neurons were distributed between 0 Hz and 250 Hz. The initial weights were chosen from the uniform distribution in the range [0, 1] for the feed-forward connections, the weights for the lateral inhibitory connections were initialized to –1, and the initial reciprocal connection weights were set to 0.

### 2.5. Hyperparameter search

The proposed learning algorithm contains a sufficiently large number of hyperparameters. Here we briefly provide the strategy chosen for their search. The parameters *T*_*input*_, τ_*m*_ and τ_*ref*_ were initially set to their values and were not changed during hyperparameter search. *I*_*supervised*_ injected into a target neuron was set to the value that was slightly greater than that guaranteeing 100 % accuracy during training. Learning rate parameters α, β, γ, η should be chosen similar in their values. Firstly, we found α = 0.1 that provides the fastest convergence to the best result for the feed-forward SNN. We then noticed that slightly greater β and η accelerated the convergence at the early training phase. The time constant of the weight decay τ was chosen to be small enough to let the hidden neurons learn zero weights, linking them to some border pixels that always provide zero signal. On the other hand, it was chosen to be large enough to prevent the appearance of “dead” neurons (with all zero weights) during training. The main parameters to which we applied the grid-search procedure were τ_*a*_, τ_θ_, τ_*th*_, Δ*V*_*th*_. The algorithm convergence appeared to be very sensitive to the $\frac{\tau_{\theta }}{\tau_{a}}$ ratio: when it became < 5 or >15, the network accuracy fell to about 10%. Also, it is very important to balance the currents introduced into a neuron that are provided by different types of connections. Details about the current balancing are described in Supplementary Material, section 3.

## 3. Results

### 3.1. Learning curves

#### 3.1.1. Original model case

The original network was trained in two modes: supervised and partially unsupervised. The first assumes that when the image is presented to the input layer, supervised currents are injected into the appropriate neuron of the last layer. Thus, the network can learn, according to the local rules (6)–(9), which should activate output neurons in response to the image of this class. In the partially unsupervised approach we allowed such supervised training only for a very short period of time, to pre-train the model and then let the weights change in an unsupervised way, according to the training rules described in section 2.2, without any supervised current injection. The response of the network was calculated as the argmax function of the vector of instant activities of 10 output neurons, in the 50–100 ms interval of the image example presentation (200 ms overall, the last 100 ms is a silent signal). Threshold dynamics were highly correlated with activities, and can therefore be used to calculate the response of the network as well.

**Figure 3:**
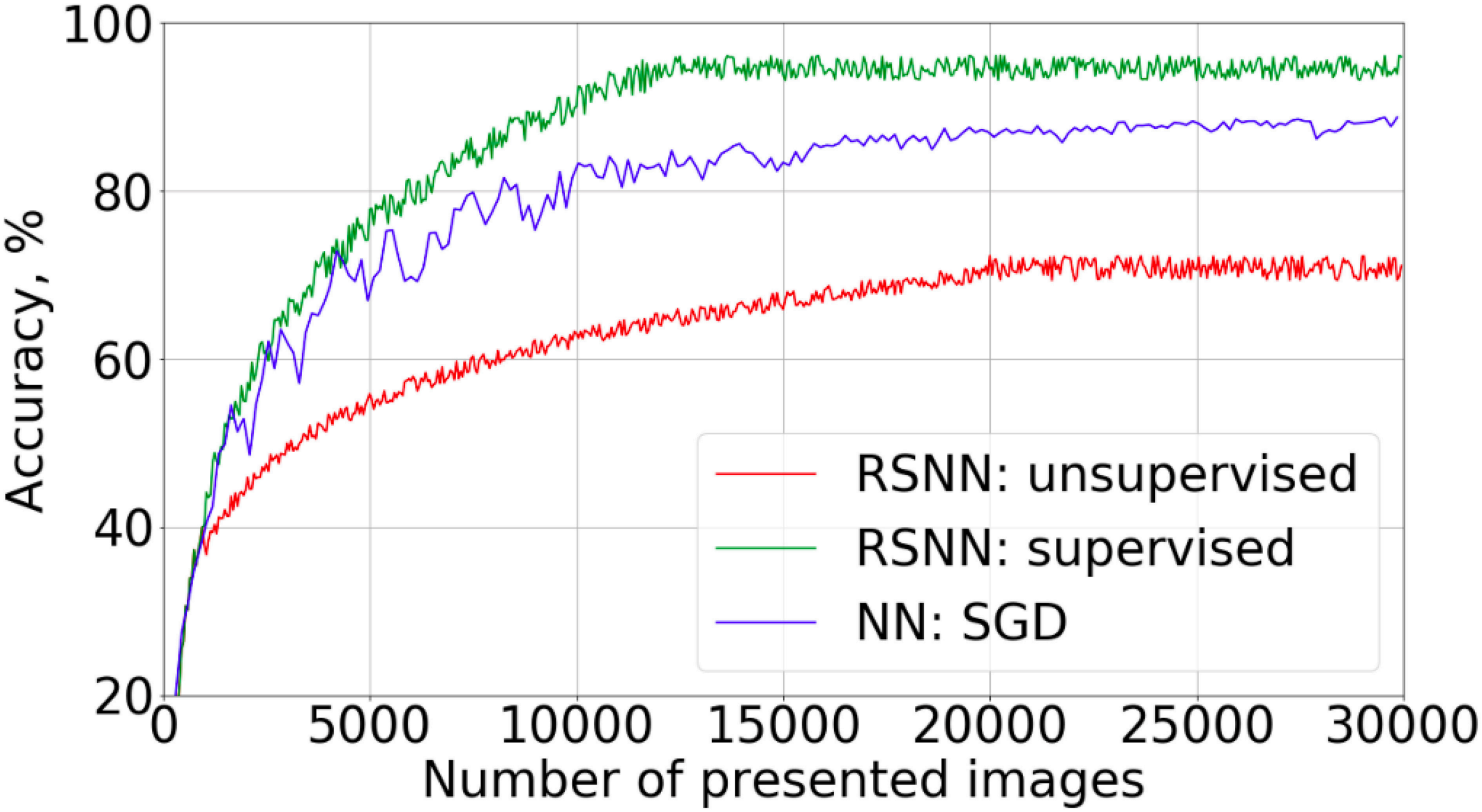
Learning curves on MNIST dataset present the recognition accuracy on the test set for the supervised mode (green), the partially unsupervised mode (red). The latter implies the full image presentations for a few images (400 in this example) at the beginning of the training in the supervised mode (with a teacher's current), followed by training without a supervised current. Learning curve for the feed-forward formal neural network (blue) with “784−100−10” architecture is presented for comparison of the convergence speed.

The learning curves for the supervised and partially unsupervised cases are shown in Figure 3. To evaluate the recognition accuracy, 10,000 images from the test set were presented to the SNN with fixed weights after every 50 training images. We were training the SNN only on 30,400 train examples to get the curves of Figure 3 because of the accuracy saturation effect. After feeding the first 400 training objects to the network we continued learning in different modes: with and without supervised currents. The best accuracy was 95.4% and 72.1% for the supervised and the partially unsupervised learning, respectively. These are not very impressive values, but the new algorithm is quite far from the optimal realization, which should be taken into account (see also section 4). We also tried to increase the number of neurons in the hidden layer up to 400 without additional adjustment of hyperparameters. An accuracy of 96.2% was obtained. We also trained a feed-forward formal neural network (perceptron) with 100 neurons in the hidden layer, to compare the convergence speed. We used an SGD algorithm (Bottou, 1998) with a learning rate of 0.1, batch size 50, ReLU non-linearity function, and acquired 98.5% accuracy after 10 epochs of training. However, the convergence speed was much slower than for supervised SNNs.

One may note the fast convergence of the FEELING algorithm. Indeed, the accuracy saturation was observed after only 12,000 images from the train set, whereas a traditional learning algorithm such as backpropagation with stochastic gradient descent, converges only after hundreds of thousands of image presentations.

#### 3.1.2. Ablation study

**Figure 4:**
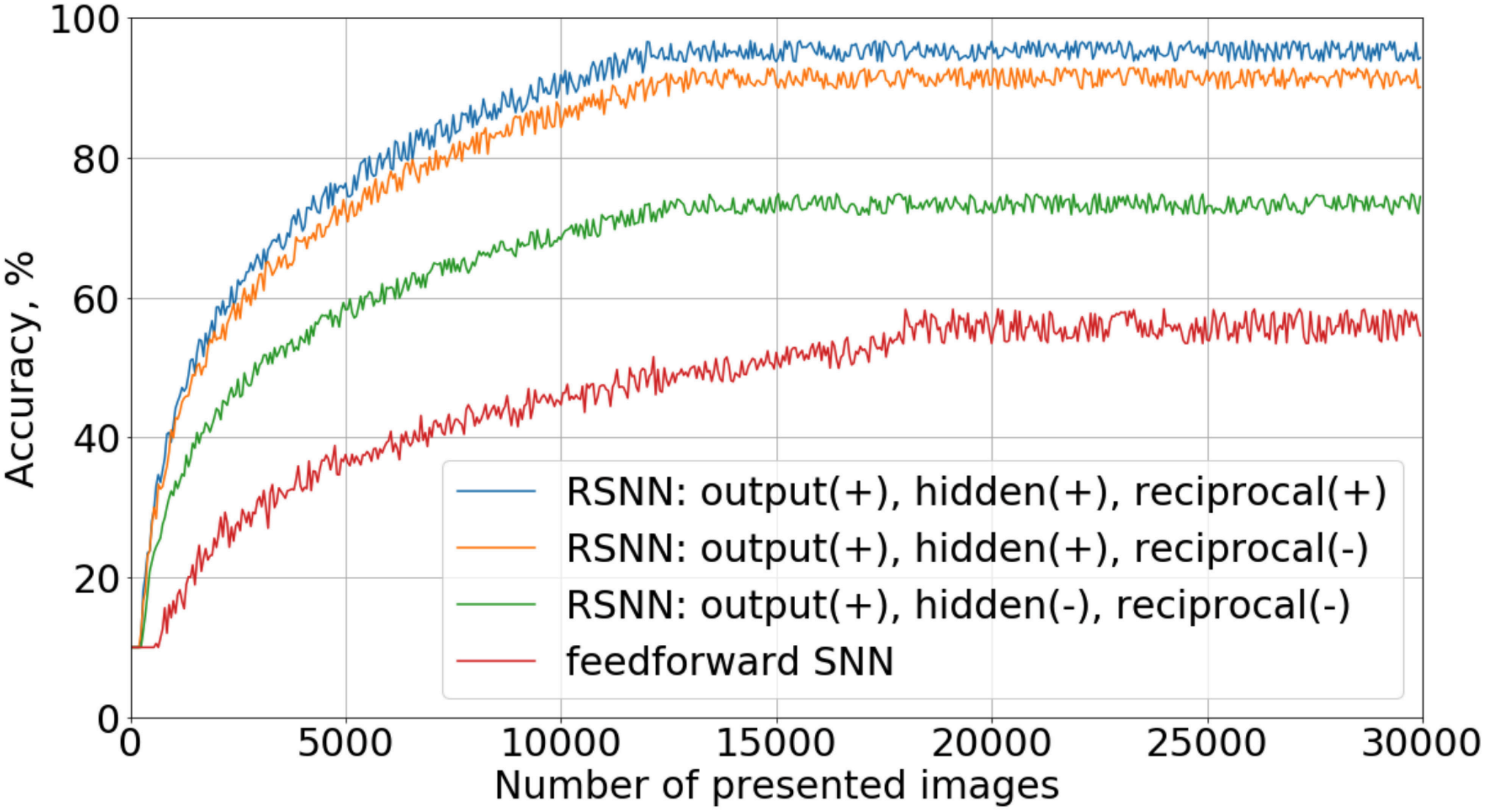
Ablation study. Learning curves for different connectivity architectures are presented. In the legend, “output(+)” means the presence of inhibitory connections in the output layer, “reciprocal(−),” the absence of reciprocal connections from the output to the hidden layer, etc.

To verify the importance of different connections in the proposed recurrent SNN architecture, we studied the contribution of each type of synapses in the network. Four types of architecture with the same number of neurons were trained with a teacher signal: a simple feed-forward network, a recurrent network with inhibitory connections only in the last layer, a recurrent network with inhibition in both hidden and output layers, and a fully connected network with the original architecture described in section 2.1.1. Note that hyperparameters such as α, β, γ, η, τ_*a*_, τ_θ_ were different from the values in Table 1 because they were adjusted separately for each type of SNN architecture to reach the best recognition accuracy results in each case. Learning curves for these experiments are shown in Figure 4. Here we evaluated our models on a test set of 10,000 images after 50 training images passed through the network. A positive effect of adding recurrent inhibitory and reciprocal connections was observed. The addition of lateral inhibitory connections provided the greatest increase in accuracy.

We do not show the results of the cases—using the notation of Figure 4 —“output(−), hidden(−), reciprocal(+)” and “output(+), hidden(−), reciprocal(+),” because they did not provide any improvement relative to “feed-forward SNN” and “output(+), hidden(−), reciprocal(−)” topologies, respectively. Without inhibitory connections in the hidden layer, neurons do not form groups, so reciprocal connections became ineffective in our experiments. Indeed, if the most active output neuron supports the weak neurons of its family, this group becomes stronger and competitively suppresses the activity of other groups in the hidden layer, and the recognition accuracy increases.

### 3.2. Visualization of neurons' receptive fields

#### 3.2.1. Output layer

**Figure 5:**
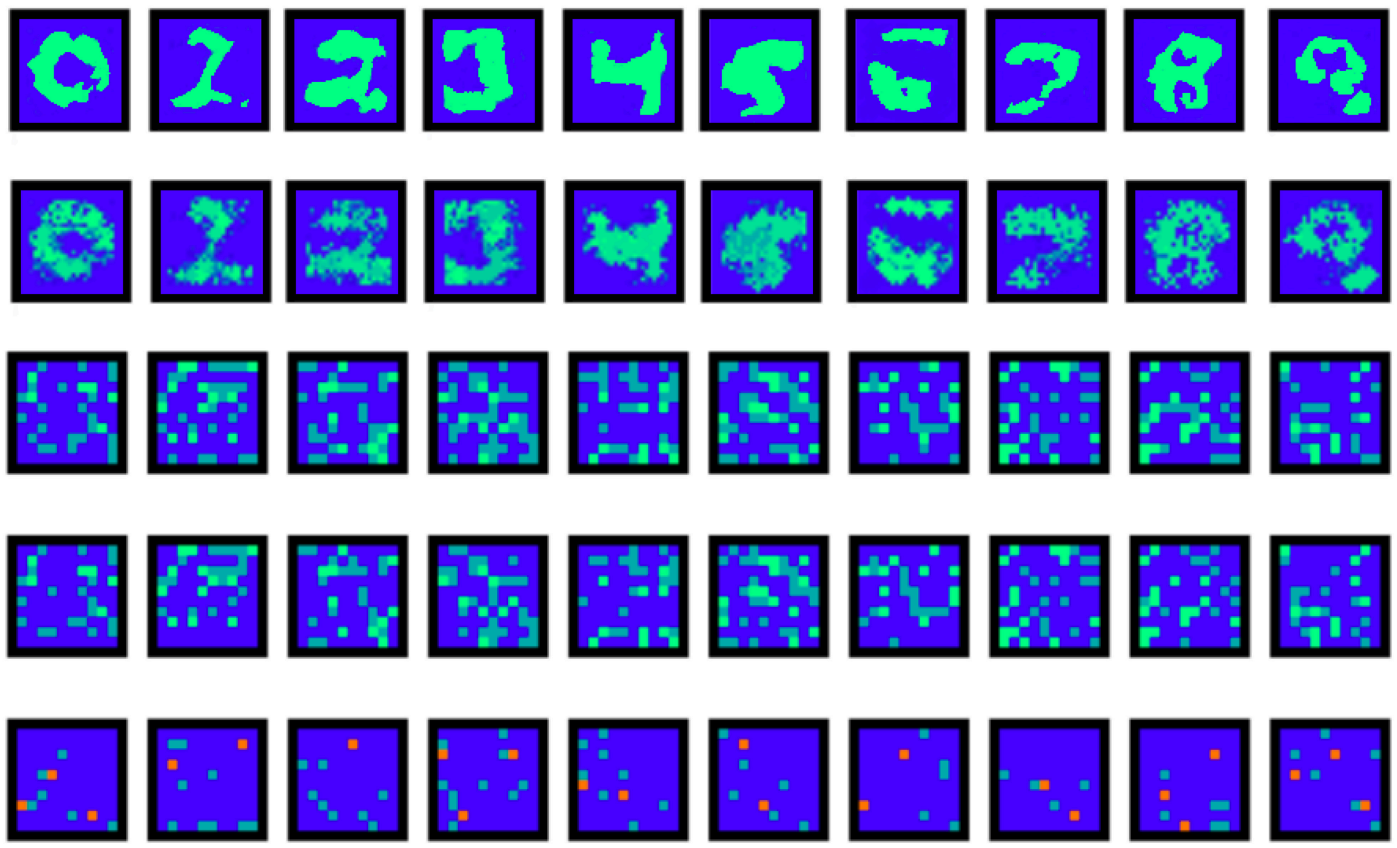
Last layer weights visualization. The first row contains reconstructed maximizing images for the output neurons (Nekhaev and Demin, 2017). The second row is a simple product of two forward weight matrices: one of the size 784 × 100 and the other of size 100 × 10. The third and the forth rows are visualization of the forward and reciprocal 100 × 10 weights. The last one is their difference: positive (green dots) and negative (red dots) values are shown.

When a neural network model is trained it is often interesting to visualize neurons' receptive fields to find out if there are some neurons that obtain label-specific behavior of their activity. It is also useful to create visualizations to verify if the initial hypothesis of maximizing neuron activities is presented in the trained network. For example, it is reasonable to check if the forward and reciprocal 100 × 10 weight matrices correlate. This analysis for the original SNN trained with the supervised algorithm is shown in Figure 5.

It is worth noting that the simple multiplication of feed-forward weight matrices gave quite recognizable visualizations of digits learned by the output neurons (the 2nd row in Figure 5). Slightly better and less noisy results were obtained by the method described in Nekhaev and Demin (2017) (the 1st row in Figure 5). It was also shown that the forward and reciprocal weights between the hidden and output layers were highly correlated. Moreover, removing the reciprocal connections after training (making their weights equal to 0) decreased recognition accuracy drastically from ~90 to ~55 %. It means that an output neuron of a certain class strongly cooperates with the hidden neurons from its family.

#### 3.2.2. Hidden layer

**Figure 6:**
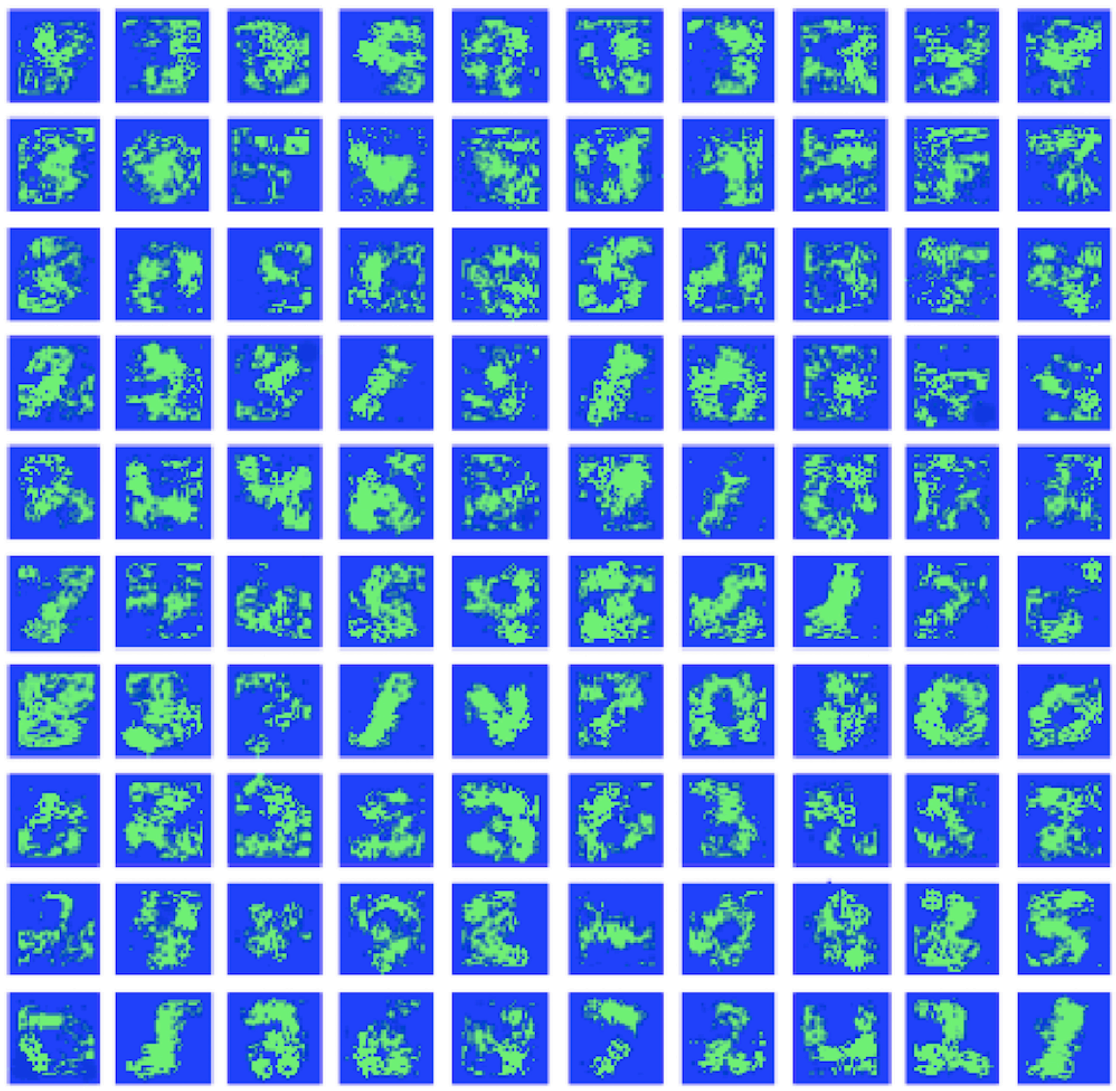
Hidden layer neuron visualization. The feed-forward weight values from the input to all 100 neurons of the hidden layer (organized here into the square 10 × 10 for convenience).

**Figure 7:**
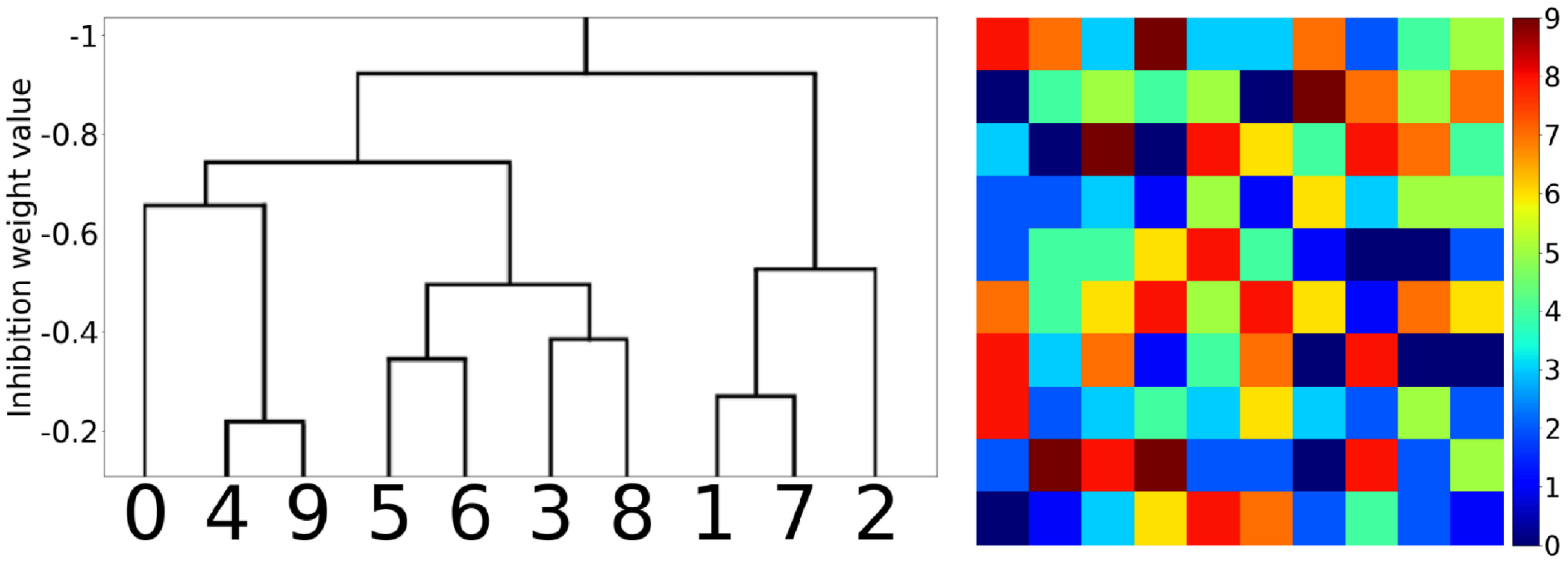
Hierarchical clustering of the neuron families. **(Left)** The tree of family clusters built by a level of competition between them, which is reflected by the magnitude of negative weights between the corresponding hidden layer neurons (marked on the vertical axis). **(Right)** 10 clusters corresponding to the minimum level of competition between hidden neurons (the cut-off weight value was chosen equal to –0.2).

The hidden layer neurons can be visualized simply by the weight values of their connections to the corresponding pixels of the input layer (Figure 6). Hidden neurons specific to one MNIST digit class (e.g., to “0”) can be joined into one family. Those families are induced during training and then compete with each other by negative lateral connections inside the hidden layer. The local rule (9) also admits cooperation between neurons belonging to the same class, i.e., a decrease in the absolute value of negative weights between them. So, it was interesting to see how those families of neurons and their associations correlated with each other after training the whole network. In Figure 7, the tree of neuron families is presented, which was built in the following way. We considered a hidden layer as a graph with 100 nodes, having connections between them, all nodes connected with each other. Each connection had a corresponding value (inhibitory weight). We sorted connections according to their values and started to prune connections with the most inhibitory weights (starting from –1). When we reached the value –0.9, two independent sub-graphs were formed (there is no single connection between neurons in two different sub-graphs) that can be seen at the top of the left-hand side of Figure 7. When we continued this procedure up to the value –0.2, ten independent sub-graphs appeared. We then mapped these sub-graphs to digit labels: visualizations of weights from the input to the hidden layer were easily associated with certain digits. Therefore, the more negative the weights between neurons were the further apart the corresponding family clusters at the hierarchical tree were. It is interesting to note that clusters were formed at the intuitive idea of visual resemblance of different digits' images. The clusters resolved at the minimum level of competition (the cut-off weight value is –0.2) are presented on the right-hand side of Figure 7. It is possible to cluster neurons into the digit classes using information about the whole weight vector of a neuron including its recurrent connections, but the quality of such an algorithm is slightly worse (see Supplementary Material, section 4) compared to the simple procedure presented here and using only lateral weights.

## 4. Discussion

The simple new local training rules for complex SNNs are based on the biologically plausible hypothesis that each neuron sets itself the goal to maximize its activity. These rules induce the formation of neuron groups with the elements cooperating inside each group and competing with neurons of other groups. Nevertheless, due to the analog values of the negative weights, there is no clear separation between groups, and a number of “marginal,” poorly specialized neurons appear. Thus, the groups should rather be called “families” meaning the “Romeo and Juliet” effect for the marginal neurons. This new property might be very important, as it builds an hierarchical structure of neuron families, corresponding to the consistent hierarchical tree of input pattern features. The similar features excite a specific group of neurons, slightly different ones activate other neurons, but these groups can join if those features are applied to the input simultaneously, while suppressing the groups corresponding to rather different features. For example, if one feature is responsible for the nose, another for the eyes, and a third one for the mouth, then together they are responsible for the face. Neuron groups corresponding to these different features are combined when a face image is applied to the input, forming one family. In our case of simple MNIST task solving, it was seen from the hierarchical clustering of neuron families that were responsible for visually similar digits (Figure 7, left).

The most successful feed-forward deep neural networks (DNN) today, operate by a similar principle, but they do not have such a flexible competition between the features inside the layers of hidden neurons. It can be assumed that this leads to the multiple repetition of neurons responsible for the same features, especially in the shallow layers (that are closer to the network's input). We therefore believe that exploration of the deep recurrent SNNs with a hierarchical neuron family structure, will significantly reduce the number of layers and/or neurons in the layers in comparison with DNNs. A similar property was introduced at the early stage of neuroscience and ANN development by different authors (Watanabe, 1960; Barlow, 1961). This was referred to as “Sparse Distributed Representation” (SDR) according to the hypothesis of sparse neuron representations of different poorly correlated features and their combinations composing objects (Foldiak, 1990; Hinton and Ghahramani, 1997; Olshausen and Field, 2004). Despite the superficial resemblance of the suggested FEELING algorithm's results to those of the SDR they are essentially different. Indeed, SDR is generally realized by a strong competition of similar or strongly correlated features and by weak or a lack of competition between poorly correlated or independent features. It could be obtained by learning with a local anti-Hebbian weight update rule of the following form [in terms of formula (9)] (Foldiak, 1990):

(11) $$\begin{array}{l} \frac{dw_{ij^{\prime }}}{dt}\propto -\epsilon \left(a_{j}a_{j\prime }-p\right), \end{array}$$

where ϵ is a positive learning rate, *p* is a constant or a slowly (compared to the neuron activity) changing value, *a*_*j*_ and $a_{j^{\prime }}$ are the activities of laterally interconnected neurons. It was shown that the inhibitory connection strengthened when neurons were jointly active, i.e., for similar or strongly correlated features. In contrast to the FEELING algorithm, it was the opposite case–cooperation [weakening of negative connections according to (9)] of neurons responsible for similar or correlated features of an object followed by the main principle suggested here, i.e., that of maximizing neuron activity. We suppose that the FEELING approach has a few important advantages compared to SDR:

1. The strategy of coding a feature by one neuron in the case of SDR is unstable, because of the possibility of accidental loss or death of this neuron. The copying of similar features by the FEELING algorithm in different neurons is a more robust information coding strategy.
2. If the number of neurons is greater than the number of uncorrelated features, then the SDR learning leads to the appearance of neurons with all zero (or close to zero) input weights (Hinton and Ghahramani, 1997). Therefore, learning new features supplied to the network's input is very inefficient, due to the absence or the weak activation of neurons. It is not the case for FEELING, because all existing neurons learn the features supplied, with hierarchical discrimination of families corresponding to uncorrelated features. A new stimulus to the network then activates some neurons, and re-learning takes place.
3. Sequential learning of independent features and their combination is incorrect in the case of SDR. To show this, assume we have a network that is learning two independent features–e.g., “O” and “x.” We then apply their combination–⊗ to the input. The SDR learning will lead, according to the joint activation of neurons responsible for initially uncorrelated features, to the formation of a strong inhibitory connection between these neurons. Eventually, this will lead to the fact that there will be only one neuron responsible for ⊗. Then, the separate features “O” and “x,” applied to the network, will be misclassified as “⊗” (perceptual inference error). This is not the case for the FEELING algorithm. By repeating the described learning procedure, we will still obtain separate neurons responsible for the features “O” and “x” but joined into one family (with zero connection between them).

Despite the FEELING's advantages shown, it would be desirable to preserve the property of representation sparsity due to its energy efficiency and bio-plausibility (Olshausen and Field, 2004). It turns out to be true for the more advanced FEELING algorithm, because of the ability to arrange spatial sparsity of neuron connectivity (then only a small part of neurons from one family is active – that is close to the activated pattern in the layer below) and due to competition between uncorrelated features (e.g., the family for “O” competes with that for “x,” so the neurons with the weakest activities from both families are inhibited by each other). Moreover, the competition between uncorrelated features, as opposed to SDR, cleared the representation code from possible background noise that was present at the input but not in the focus of the network, i.e., with the neuron activity insufficient for competition with the main objects in the focus.

The learning algorithm demonstrated in this work allows one to build a hierarchy of features by local weight update rules, potentially not requiring the huge labeled training databases, because of the capability of unsupervised learning after the relatively short period of supervised learning.

To the best of our knowledge, this work is the first unambiguous demonstration of the hierarchical family tree construction due to the flexible cooperation and competition inside the hidden layer of neurons in an SNN. The simple method of by-intralayer-weight clustering shown above can be used for a more subtle analysis of the network topology formed. In its turn, it may be useful for engineered re-connection or re-distribution of the weights between neurons in future SNN learning techniques. Moreover, this could be the way to model *in silico* the mechanisms of formation of different nuclei, ganglia and other structures in a biological nervous system, due to their functional properties and inter-relations. Of course, the directed research is necessary to confirm or reject the possibilities described here.

The proposed “Family-Engaged Execution and Learning of Induced Neuron Groups” or, briefly, the FEELING algorithm can evidently work with different architectures of SNNs, with all types of connections between neurons, with fast convergence, and can operate in supervised as well as unsupervised modes. However, FEELING, in its current form, has one serious drawback—the MNIST benchmark accuracy and recognition is not high enough, especially as a result of partially unsupervised training. Comparison of our proposed approach for training SNNs with other methods is shown in Table 2. Our algorithm achieves the highest accuracy (by supervised learning) among two layer networks with local training rules, but convolutional networks and backpropagation algorithms still outperform the proposed method. So, applying the FEELING algorithm to a convolutional architecture seems to be a good step forward. It is also worth noting that an increase in the number of neurons in the hidden layer will likely improve the SNN accuracy, as a result of (i) a better division of similar input features in the space of higher dimensionality, and (ii) reduction of statistical spread in the number of neurons and their connectivity strength (roughly, the total sum of the neuron input weights), responsible for different classes.

**Table 2:** Recognition accuracies of the proposed training algorithm and others on the MNIST dataset. Rate-based neural coding means the spike-based network with the rate coding.

**Architecture**	**Hidden layers**	**Neural coding**	**Learning-rule**	**Accuracy (%)**
Dendritic neurons (Hussain et al., 2014)	-	Rate-based	Morphology	90.3
Convolutional SNN (Zhao et al., 2015)	Convolutional coding	Spike-based	Tempotron	91.3
Spiking RBM (O'Connor et al., 2013)	500-500	Rate-based	CD	94.1
Two layer network (Diehl and Cook, 2015)	100	Spike-based	STDP	82.9
Two layer network (Diehl and Cook, 2015)	6400	Spike-based	STDP	95.0
Two layer network (Lee et al., 2016)	800	Rate-based	Back-prop	98.56
Three layer network (Lee et al., 2016)	300-300	Rate-based	Back-prop	98.71
Convolutional SNN (Lee et al., 2016)	conv(20)-conv(50)-200	Rate-based	Back-prop	99.3
Convolutional SNN (Kheradpisheh et al., 2017)	conv(30)-conv(100)-100	Spike-based	STDP	98.4
Two layer network (ours)	100	Rate-based	FEELING	95.4
Two layer network (ours)	400	Rate-based	FEELING	96.2

At the same time, this is the first demonstration of the algorithm and, most likely, is far from optimal. The possible sources of improvement could be: (i) other local training rules for the weight update, as we have used just one of the numerous alternatives from those which could be computationally economic, reflecting the main principle of maximizing neuron activity, and biologically realistically; (ii) more appropriate weight initialization before training, to make competition between neurons more gentle and equitable; (iii) other architecture and topologies of SNNs, e.g., the addition of separate inhibitory neurons instead of the biologically implausible inhibitory connections of excitatory cells, implementation of sparse topology of a network's connectivity, variation of the number of neurons and layers. Lastly, different tactics of learning should be tested, i.e., with alternating periods of supervised and unsupervised training, with different sources and parameters of noise, and others. Thus, the FEELING algorithm can be further developed both to meet the requirements of various practical tasks and to be employed in fundamental research with more complex bio-inspired architectures of SNNs.

## Author contributions

VD and DN developed the theory and wrote the paper, DN performed the experiments.

### Conflict of interest statement

The authors declare that the research was conducted in the absence of any commercial or financial relationships that could be construed as a potential conflict of interest.
