# Supplementary material for: Recurrent Spiking Neural Network Learning Based on a Competitive Maximization of Neuronal Activity
Source: Front Neuroinform. 2018 Nov 15;12:79. doi: 10.3389/fninf.2018.00079 (PMC6250118; doi:10.3389/fninf.2018.00079)
Supplement: Supplementary file 1 [file Data_Sheet_1.PDF]

Supplementary Material:
Recurrent Spiking Neural Network Learning Based on a Competitive Maximization of Neuronal Activity

1 NOISE IN THE WEIGHT UPDATE RULES

We noticed that once the parameters τ_a and τ_θ are chosen, there an interesting feature arises. The proposed weight update rules depend on difference between instant and average firing rates for all types of connections. If we look at instant $a(t)$ and average $\theta(t)$ firing activities of a neuron in the input layer under constant firing probability, we expect to see zero difference $(a(t) - \theta(t))$. According to Poisson temporal distribution of spikes the difference of firing rates $(a(t) - \theta(t))$ is non-zero. Moreover, its expected value converges to zero only for a long period of simulation that is more than $\sim 3\tau_\theta$. The distribution of this residuals is Gaussian (Fig.S1), so we interpret this effect as the Gaussian noise inside the synapses during training. The parameters of this probability density function are determined by the time constants τ_a and, to a lesser extent, τ_θ. Generally, the larger τ_a, the smaller the standard deviation. We believe that this Gaussian noise of Poisson nature helps SNN to avoid the local minima of its loss function during training, but we did not aim to investigate this question thoroughly in this work. It should be the subject of the future research.

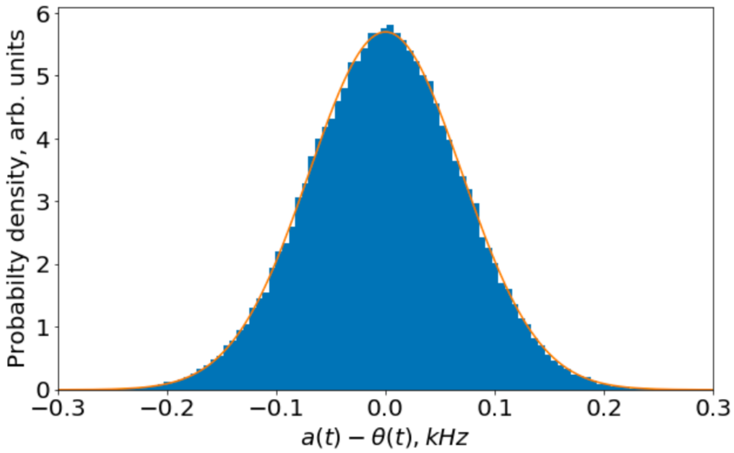

Figure S1: The stationary distribution of a time-series of the difference between instant and average activities of a neuron at a constant firing Poisson probability (300 Hz rate). It can be considered as a Gaussian noise with the zero mean in synapses during training. The Gaussian curve (orange) with zero mean and $\sigma = 0.7$ is fitted to the distribution of $(a - \theta)$.

2 CONVERGENCE OF LEARNING BY BCM-LIKE RULES

Different kinds of BCM-like rules, although subject to the maximization activity principle, can have quite different possibilities for learning algorithm's convergence. Here, we compare 2 alternatives of BCM kind rate-dependent rules for the weight update between pre-synaptic neuron i and post-synaptic neuron j:

$$\frac{dw_{ij}}{dt} \propto \delta(t - t_j)(a_i - \theta_i), \tag{S1}$$

$$\frac{dw_{ij}}{dt} \propto \delta(t - t_i)(a_j - \theta_j). \tag{S2}$$

For the sake of simplicity, we do not consider the term $-w_{ij}/\tau$, describing the rather slow relaxation of weight to 0. With this disclaimer, (S1) corresponds to the formula (6) of the main text, and (S2) reflects the original BCM rule, accurate to qualitatively inessential for the simulation factor a_j (see below). It should be noted that the term $\delta(t - t_i)$ ($\delta(t - t_j)$) means the weight update by the spikes of pre-synaptic (post-synaptic) neuron, and thus is almost equivalent to the rate-coded activity a_i (a_j), when considering equation (S1) ((S2)) in the continuous time. The designations in (S1) - (S2) are the same as in the main text of the article.

Let see how the training of a neuron occurs when applying (S1) or (S2) rules. Assume that a neuron is specialized for recognizing the digit “7” (own class). Then it reacts also on the visually similar class “1” (but weaker than to “7”) and almost does not respond to the class “0”. Therefore, we expect the following weight updates of connections with high rate-coded and low rate-coded pixels (pre-synaptic neurons), shown in Table S1. Low activity pixels represent not only background completely dark pixels but also the ones with low intensity surrounding the contour of an image sample. The sign ‘$\gg 0$’ means an intensive positive weight update, the ‘$>\approx 0$’ implies a positive update close to zero, ‘> 0’ indicates a moderate weight change compared to the intensive and the weak ones, and so on.

Sample class \ Weight update		$\Delta w_{ij} \propto \delta(t - t_j)(a_i - \theta_i)$		$\Delta w_{ij} \propto \delta(t - t_i)(a_j - \theta_j)$	
		High activity pixels	Low activity pixels	High activity pixels	Low activity pixels
Own class	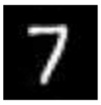	>>0	<<0	>>0	>0
Not own class	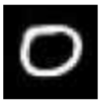	>≈0	<≈0	<0	<≈0
Interim class	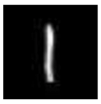	>0	<0	>≈0	>≈0

Table S1. The weight updates mediated by two BCM-like rules. The designations are described in the text of this section.

It can be seen that the rule (S1) used in this work demonstrates the learning to highly contrast images of the own class: it strengthens the weights to the high activity pixels and weakens connections to low intensity contour pixels of an image. However, this rule tries to learn also the images of the interim and, to a less extent, of not own classes. That is why it can be called a ”greedy” high contrast algorithm.

The BCM rule (S2) has a rather different behavior. It learns the images of its own class jointly with low intensity contour and background pixels. At the same time, it does not pretend to other's property. Nevertheless, it can try to capture the interim class images. So, this rule leads to not greedy but low contrast learning.

Numerical experiments have shown that the high contrast is more important than the greed of an algorithm. First, (S2) rule captures the low intensity pixels around the images of its own class. Then it begin to respond more than in average to the interim class images and captures them too. After that, not own class goes to the category of interim classes, and the situation is repeated. Thus, the BCM-like learning by the rule (S2) is shown experimentally to be actually more greedy algorithm. Multiplying (S2) to the factor a_j, as in the original BCM rule, only aggravates the situation, because it weakens reaction of attenuation the weights with not own class images. Applying (S2) instead of (S1) rule, almost in any cases and at any values of hyper-parameters, leads to formation of a neuron group that captures all or almost all image classes (Fig. S2).

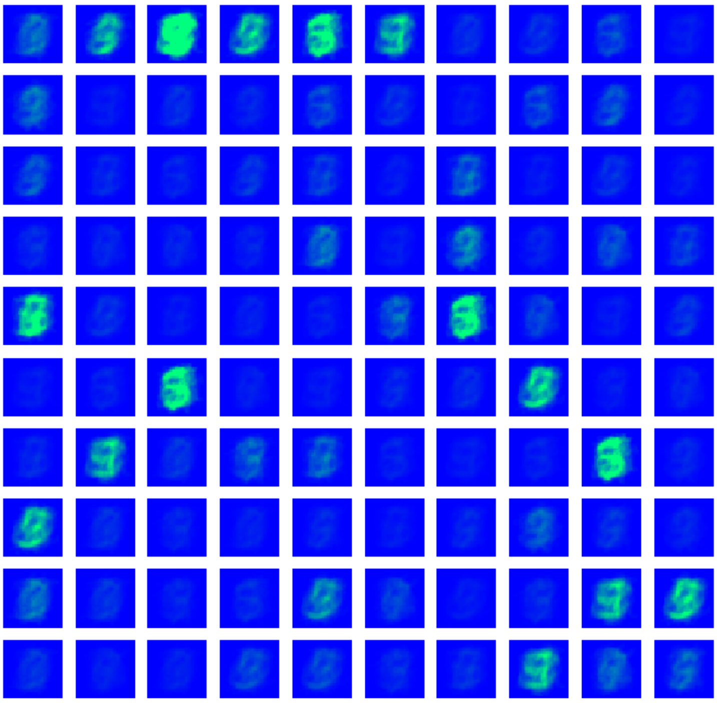

Figure S2: Typical visualizations of the weights of neurons capturing almost all image classes during learning by the rule (S2).

This is not the case for the training rule (S1). Despite the greed of this rule, the high contrast learning and equitable competition between neurons in the hidden layer leads to a clear separation of images into classes, so that a weak response to the images of not own or even interim classes does not change the current configuration of the weights.

3 CURRENT BALANCING

As it was mentioned in the main text, there is a possibility to fail the convergence of learning, with inappropriate hyper-parameter values setting. In some situations a subgroup of hidden neurons forms a strong family because of their cooperation and suppresses activity of all the other neurons. It mainly depends on the training rules' parameters, especially on the parameters responsible for the balancing of different types of currents: feed-forward, reciprocal and inhibitory. For a particular neuron the total current is computed as follows:

$$I_{total} = w_{ff} I_{ff} + w_{inh} I_{inh} + w_{rec} I_{rec} \quad \text{(S3)}$$

Most of the time in hyper-parameter adjusting was spent on the balancing of the coefficients w_{ff}, w_{inh}, w_{rec} to ensure the equitable competition between neuron groups. As a result of a grid-search procedure, the values of $w_{ff} = 0.3$, $w_{inh} = 1.7$, $w_{rec} = 2.5$ were found to be near optimal for the current balancing. With this set of parameters, the contribution of the forward current to the I_{total} is 50%, 35% for inhibitory and 15% for reciprocal currents on average. Video with a visualization of weight updates during training is available online [1]. The simple product of two weight matrices 784×100 and 100×10 is presented on each frame of the video. The first one demonstrates evolution of the network weights during 5000 ms (250 input images) with current balancing coefficients values mentioned above. In the second video, the network was trained during 50000 ms (2500 input images). It demonstrates the effect of unequal competition ($w_{ff} = 0.3$, $w_{inh} = 2.5$, $w_{rec} = 0$) that results in formation of the weak neuron groups responsible for the digits ”4”, ”5” and ”9”.

4 FULL WEIGHT VECTOR NEURON CLUSTERING ALGORITHM

Clustering shown in Fig.7 of the main text had been done using only lateral inhibitory weights of the hidden neurons. It is interesting to see if there will be some improvement of clustering neurons using additional information about all other weights of a hidden neuron (its 784 input connections, 100 lateral weights (self-connection is assumed to be 0), and 10 output feed-forward connections to the classifying layer). It was done for the preprocessed full vectors of weights for hidden layer neurons by k-means algorithm with 10 clusters. Preprocessing was the dimensionality reduction from 894-dimensional weight vector to 10 top dimensions by the Principal Component Analysis (PCA) algorithm.

It can be seen that there is a good coincidence of this cluster presentation in Fig.S3 to the clusters resolved at the minimum level of competition (the cut-off weight value equals to –0.2), which are presented at the right-hand side of the Fig.7 of the main text. Moreover, the analysis of the subtle differences (e.g., the 6th, 9th and 10th neurons in the last row of both clustering square diagrams) shows (compare classes of these neurons according to both clustering diagrams with their receptive field images in Fig.6 of the main text) that the first simple way of clustering (using only lateral weights) is more appropriate than the complex mathematical k-means-followed-by-PCA algorithm.

[1] https://www.youtube.com/channel/UCHB2xeQ6zzlXyW9ImMnFo1g

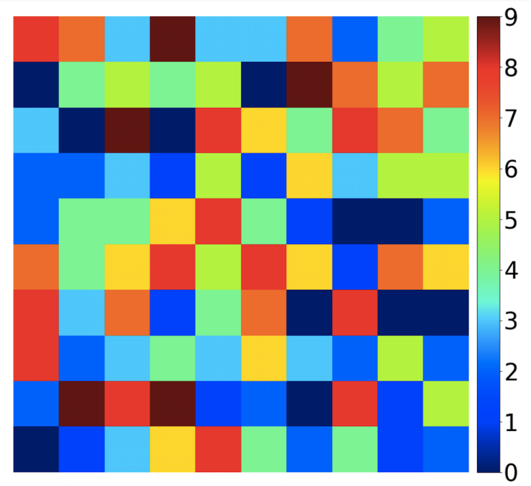

Figure S3: The clusters of hidden layer neurons obtained by the application of k-means-followed-by-PCA algorithm to the full weight vectors of neurons and corresponding to the different classes of digits marked by colors (cf. the legend).
